# 

**Published:** 1966-01

**Authors:** 


### Contents

SOCIETY, ARTISTS, AND THE PLAGUE *R. E. Hemphill* 1

MILK AND TUBERCULOSIS *A. D. Osborne and A. V. Neale* 12

UNILATERAL RENAL ARTERIAL OBSTRUCTION
*Clinico-Pathological Conference* 15

NOTES AND NEWS . . . . . . . . . . 21

